# Encountering aged care: a mixed methods investigation of medical students’ clinical placement experiences

**DOI:** 10.1186/s12877-016-0211-8

**Published:** 2016-02-04

**Authors:** Michael J. Annear, Emma Lea, Amanda Lo, Laura Tierney, Andrew Robinson

**Affiliations:** Wicking Dementia Research and Education Centre, Faculty of Health, University of Tasmania, Private Bag 143, Hobart, TAS 7001 Australia; School of Medicine, Faculty of Health, University of Tasmania, Private Bag 34, Hobart, TAS 7001 Australia; School of Health Sciences, Faculty of Health, University of Tasmania, Private Bag 135, Hobart, TAS 7001 Australia

**Keywords:** Medical students, Aged care facilities, Clinical placement, Older adults

## Abstract

**Background:**

Residential aged care is an increasingly important health setting due to population ageing and the increase in age-related conditions, such as dementia. However, medical education has limited engagement with this fast-growing sector and undergraduate training remains primarily focussed on acute presentations in hospital settings. Additionally, concerns have been raised about the adequacy of dementia-related content in undergraduate medical curricula, while research has found mixed attitudes among students towards the care of older people. This study explores how medical students engage with the learning experiences accessible in clinical placements in residential aged care facilities (RACFs), particularly exposure to multiple comorbidity, cognitive impairment, and palliative care.

**Methods:**

Fifth-year medical students (*N* = 61) completed five-day clinical placements at two Australian aged care facilities in 2013 and 2014. The placements were supported by an iterative yet structured program and academic teaching staff to ensure appropriate educational experiences and oversight. Mixed methods data were collected before and after the clinical placement. Quantitative data included surveys of dementia knowledge and questions about attitudes to the aged care sector and working with older adults. Qualitative data were collected from focus group discussions concerning medical student expectations, learning opportunities, and challenges to engagement.

**Results:**

Pre-placement surveys identified good dementia knowledge, but poor attitudes towards aged care and older adults. Negative placement experiences were associated with a struggle to discern case complexity and a perception of an aged care placement as an opportunity cost associated with reduced hospital training time. Irrespective of negative sentiment, post-placement survey data showed significant improvements in attitudes to working with older people and dementia knowledge. Positive student experiences were explained by in-depth engagement with clinically challenging cases and opportunities to practice independent clinical decision making and contribute to resident care.

**Conclusions:**

Aged care placements can improve medical student attitudes to working with older people and dementia knowledge. Clinical placements in RACFs challenge students to become more resourceful and independent in their clinical assessment and decision-making with vulnerable older adults. This suggests that aged care facilities offer considerable opportunity to enhance undergraduate medical education. However, more work is required to engender cultural change across medical curricula to embed issues around ageing, multiple comorbidity, and dementia.

## Background

The medical profession has been challenged to engage more effectively with older adults [1, 2]. Echoing global trends, the proportion of Australia’s population aged 65 and older is predicted to increase from 13 % in 2007 to 23 % in 2061 [3]. This cohort makes up the highest proportion of those seeking medical services – comprising 28 % of general practice visits in 2010 [4, 5]. The challenge for the medical profession is to inculcate undergraduate interest in aged care in the context of an increasing prevalence of older people suffering chronic illness and multiple comorbidities.

While medical professionals from most specialities will encounter older patients, shortcomings have been noted in undergraduate curricula worldwide with regard to content about older-adult health [2]. Evidence suggests that management of acute illness associated with hospitalisation dominate medical curricula [6, 7]. For example, a United Kingdom survey identified a lack of teaching time in medical undergraduate curricula related to ageing, where less than two weeks of a five-year degree addressed health care for older people [8]. Across Australia, only 0.5 % of medical students’ clinical placement hours are undertaken in residential aged care facilities (RACFs – also referred to as nursing homes), compared to 78 % of clinical placement time in hospitals [9]. RACFs in Australia provide a mix of high and low care as well as community outreach services for older people with cognitive and physical impairments (often multiple comorbidities) who require specialised care. These facilities are administered by private and not-for-profit organisations, with government funding support, to meet the needs of individuals who have been assessed as requiring institutional care. Limited medical student engagement with chronically unwell older adults may be exacerbated by a lack of interface between acute care and RACFs [10] and the absence of an established medical presence in these settings [11].

Concerns have also been raised about the adequacy of dementia-related content in undergraduate medical curricula [12]. In the context of an increasing prevalence of dementia associated with population ageing [13], educationalists from Australia and the United Kingdom have recommended expanding opportunities for experiential learning and skill development regarding dementia [12, 14]. Current curricular deficiencies are likely to have a negative impact on students’ knowledge of and attitudes towards geriatric medicine [15, 16] and their capacity to effectively engage with cognitively impaired older adults [17].

Compounding curricular shortcomings are the mixed attitudes held by medical students towards care of older people [18–22], including what Higashi et al. [19] describe as persistent and implicitly negative attitudes. Persistent negative student attitudes towards aged care and older adults may be due to an educational culture that reinforces negative stereotypes about older adults [18], communication challenges that limit reciprocal and effective patient-centred relations [23], social distance between learners and RACF residents that reduces empathy [24], low levels of health literacy and compliance [24], or stark differences from the hospital training environment [25].

Despite prevailing negative sentiment, evidence suggests that RACFs provide an ideal setting for medical training and exposure to frail older people with complex health problems, including dementia [26, 27]. In Australia, for example, 52 % of permanent RACF residents had a recorded diagnosis of dementia in 2011 [28], coexisting with an average of six long-term comorbidities [29]. As the majority of aged care is provided in the community rather than hospitals [30], RACFs are a particularly appropriate setting for facilitating medical student engagement with aged care and frail older adults, many of whom have dementia.

There are a small number of published reports of undergraduate medical student RACF clinical placements in Australia and the United States [31–33]. Clinical placements refer to one-off and embedded learning experiences for undergraduate students within particular healthcare settings for the purposes of increasing understanding about and exposure to a specialised sector (i.e. aged care, primary care, ambulance). Two Australian studies have previously reported on programs that include medical students in inter-professional clinical placements at RACFs in Western Australia and Tasmania [32, 33], while a North American study evaluated mandatory geriatrics clerkships for third-year medical students [31]. These studies were limited, however, by small samples and a lack of explanatory qualitative data derived from medical students. The current study explores first-hand medical student experiences of clinical aged care placements in Australia to understand how undergraduates engage with the available learning opportunities in a fast-growing, though largely unfamiliar, sector of health.

## Methods

### Approach

This study followed a mixed methods design [34] wherein quantitative and qualitative data were collected concurrently to provide verification of the study findings. A focus of this research was to provide students with opportunities for extended and reflective responses via focus group discussions, while also employing quantitative survey measures to systematically evaluate learning outcomes.

### Setting, sampling, and recruitment

Mandatory five-day medical student placements were undertaken in two large Tasmanian RACFs (120–140 high care places) involved in the Wicking Teaching Aged Care Facilities Program (TACFP) [35] during 2013 and 2014 (with research data only collected during the first semester of 2014). Facilities were purposively selected as they were participants in the ongoing TACFP and had an enduring research relationship and memoranda of understanding with the research institution and associated School of Medicine. All medical students undertaking their final (fifth) year of training at a Clinical School in southern Tasmania participate in RACF clinical placements, initiated via the TACFP from 2012. All available students (*N* = 75) within this Clinical School were sampled and undertook one week clinical placements in the two RACFs. The medical student cohort was composed of young students who had limited previous exposure of the aged care environment and who had not previously experienced a clinical placement in this setting. Over the course of the study, 10 groups of students attended RACF placements, with 5-7 students (*n* = 61) per group (randomly assigned by the School of Medicine course coordinator). Before each clinical placement, students attended an information session about the research arm of the placement and were provided with the opportunity to give written consent to participate. The study was approved by the University of Tasmania Human Research Ethics Committee for Social Sciences (Ref. No. H0011576).

The clinical placement, and the broader Wicking TACFP, was underpinned by an iterative action research process that led participants, including 20 RACF staff who acted as mentors, through cycles of problem identification, planning, action and reflection, to improve the placement and overcome barriers to student engagement [36]. A cycle of continuous improvement was engendered within the TACFP, which meant that successive student cohorts could expect to have progressively improved clinical placement experiences. Beginning in 2012, changes that were made to the clinical placement program to improve student engagement and learning included: a) the inclusion of a formal program of inter-professional learning; b) increased focus on dementia with on-site dementia education; c) increased contact time between medical students and residents to foster comprehensive assessments; d) revision of student calendars to ensure that RACF placements did not compete with clinical placements in other settings (i.e. hospitals); and e) reliable facility mentorship and General Practitioner (GP) tutor support to guide students through learning challenges, help them to identify appropriate clinical issues, and access appropriate patient information in an unfamiliar clinical environment. Placements included clinical, non-clinical, and inter-professional learning opportunities, with a focus on collaborative assessments of volunteer residents and the presentation of resident case studies, which addressed student assessments and recommendations for improving care [35]. Up to 80 residents (across both facilities) were invited to participate in supervised and student-led health assessments by senior nursing staff at both facilities. As the assessments were not part of a research process, written consent from residents was not required and they were free to choose not to interact with students if they wished. Family members of residents were also informed about the student placements and assessments.

Oversight and assessment of medical student performance was undertaken by a GP tutor employed by the Clinical School who did not have direct responsibility for the care of RACF residents. The tutor met with the students for 1.5 hours daily and facilitated sessions where, among a range of activities, she challenged them to critically analyse their understanding of the clinical issues experienced by residents and assessment of their case presentations. When students were on the RACF wards, they were supported by registered nurses and professional care staff (referred to as extended care assistants or personal care assistants in Australia).

### Data collection

Medical students completed pre- and post-placement surveys of dementia knowledge (as a proxy measure for understanding of issues relevant to aged care) and participated in a one-hour focus-group discussion on the final placement day. The Dementia Knowledge Assessment Survey (DKAT 2), a reliable and valid measure of dementia knowledge [37], was administered in order to assess changes in student knowledge. Focus groups were convened by a researcher with training and experience in facilitated group discussions with support from a research assistant and note taker. Each focus group discussion lasted for 60 mins. Semi-structured questions addressed pre-placement expectations; differences from hospital training environments; learning opportunities and challenges; and interactions with residents, staff, and other student learners. Examples of questions asked of respondents included, a) how was the aged care placement different from other clinical placements that you have experienced during your undergraduate training, b) how, if at all, has this aged care placement affected your understanding of the health issues faced by older people, and c) has this clinical placement experience changed your level of interest in pursuing a career in geriatric medicine? Probing questions were also used to follow-up new lines of enquiry. All discussions were audio-recorded and transcribed to facilitate analysis. In most student focus groups there was a clear demarcation between those who found the clinical placement rewarding and those who felt it was not an appropriate location for medical training. This created a tense climate with much debate and deliberation about the value of the learning experience. In this context, the experience of the facilitator was crucial to ensuring that all voices were heard and diverse perspectives represented.

### Analysis

Descriptive and inferential analyses of demographic and survey data were performed using SPSS (version 20) [38]. The Wilcoxon signed rank test was used to evaluate potential changes in dementia knowledge scores and expected/actual enjoyment from the beginning to the end of the placement week. The Wilcoxon signed rank test provides a mechanism for testing whether non-linear data from a group of respondents differs significantly over two time points, usually following an intervention (clinical placements in this case). Qualitative data from 10 medical student focus group discussions were evaluated to assess their RACF placement experiences. Focus-group data were analysed using techniques commonly employed in thematic analysis of subjective reports [39]. NVIVO (version 10) qualitative data analysis software was used to facilitate the organisation and coding of data [40]. A two-step coding procedure was employed, which was previously described by Lofland and Lofland [39] and is influenced by principles of Grounded Theory Analysis [41]. Transcripts were initially examined to identify broad categorical statements (also referred to as descriptive or initial codes) relating to clinical placement experiences and interactions with older adults. Following this, analytical (focussed) coding was used to identify respondent statements that best reflected latent or underlying themes that were contained within the data. Analytic codes formed the basis of memos (summarised statements about the content of emergent ideas), which were developed into more detailed research themes (presented in the results section). This approach to coding is often referred to as *inductive qualitative thematic analysis* and is a commonly articulated approach in the social sciences and health services research [39, 42]. Emergent themes were verified by two researchers (MA and EL) who coded the data independently to improve inter-rater reliability, procedures that are consistent with standard qualitative analysis guidelines for enhancing rigor in data analysis [43]. The level of agreement between the two researchers was calculated at >95 % using the NVIVO coding comparison query. All themes were confirmed by the medical course coordinator (AL) for the fifth-year undergraduate program. Confirmation of themes involved the medical course co-ordinator reading the draft analysis in its entirety and testing theme descriptions against her experiences of coordinating the placement and discussions with medical students. The medical course coordinator did not participate in the focus group discussions and was well-placed to provide an informed perspective on the thematic descriptions that was independent of the subjective student experience.

## Results

### Cohort characteristics

During 2013 and 2014, 75 medical students attended clinical placements in the two RACFs. In total, 61 students completed the research component of the placement (81 % response rate). One student withdrew from the medical training course and did not complete the research, while 13 students did not attend the data collection and focus-group sessions as they had either not provided consent or failed to attend the discussions. In general, the medical student cohort were not satisfied when notified that they would be undertaking a placement in an RACF nor were they positively predisposed to working with older adults (see Table 1 below).

**Table 1:** Demographic and attitudinal characteristics of 2013 and 2014 student cohorts

Demographic and attitudinal information	2013 (*n* = 23)	2014 (*n* = 38)
Age (mean years and standard deviation)	25 (*SD* = 4)	24 (*SD* = 3)
Female gender	57 %	47 %
Born in Australia	83 %	63 %
English as first/main language	100 %	84 %
Prior aged care experience		
Previous work/training experience in an RACF	9 %	8 %
Previous visit to an RACF for any reason	70 %	53 %
Pre-placement attitudes towards aged care		
Satisfied when notified of RACF placement	13 %	16 %
Expect to enjoy working with older adults	39 %	39 %
Post-placement attitudes towards aged care		
Understanding of RACFs has increased	96 %	92 %
Enjoyed working with older adults	72 %	83 %

### Quantitative findings

Following a five-day RACF placement, there was a modest, statistically significant 5 % increase in dementia knowledge among the medical student cohort, *z* = -2.63, *p* = .009, with a moderate effect size (*r* = .37). At the end of the clinical placement, the mean dementia knowledge score among the medical student cohort was 19.41 out of 21.00. Despite scoring at the upper limit of the DKAT 2, medical students exhibited comparatively lower scores on items addressing symptoms and care provision: a) difficulty swallowing occurs in late-stage dementia (54 % scored correctly); b) movement is limited in late-stage dementia (59 % scored correctly); c) it is important to correct a person who has dementia when they are confused (70 % scored correctly). Across the five-day program, there was a statistically significant 37 % improvement in medical students’ expected and actual enjoyment of working with older people from the beginning to the end of the clinical placement week, *z* = -3.08, *p* = .002, with a moderate effect size (*r* = .27), indicating a large improvement in attitude. Knowledge and attitude data indicate a high level of student engagement with the RACF learning experience by the end of the placement week.

### Qualitative findings

Thematic analysis of qualitative data revealed four themes and contrasting learning experiences among the medical student cohorts across the two-year project: 1) *dementia challenges assessment competence* ; 2) *aged care is perceived as an unnecessary opportunity cost*; 3) *aged care placements stimulate problem solving and critical reflection*; and 4) *contributing to resident health reinforces training and clinical decision making*. These themes reflect a continuum of learning experiences among final year medical students who attended an RACF clinical placement.

#### Theme 1: Dementia challenges assessment competence

During the focus group discussions, student comments suggested that some struggled to engage with the relevance of the clinical placement. These students had difficulty recognising the complexity of the care needs of frail RACF residents, most of whom had multiple comorbidities and cognitive impairment. In the absence of an immediately accessible medical supervisor, these students assessed residents with limited or no reference to the opinions of facility staff, family members, or their interdisciplinary peers. When they encountered residents with dementia, they reported experiencing a sense of futility because they did not know how to gather information from non-communicative respondents. In this context, dementia was seen as compromising their capacity to undertake a medical assessment. For example, one student commented, “*I had a patient with dementia. I spent 15 minutes with her before I decided that there wasn’t much more I could say or do*” (EM5030, 2013). Another student reported,

We get told to do these comprehensive medical assessments, which are fine if you have a cognitively intact patient, but if you’ve got a patient with dementia there’s only so much you can do in this setting. One of my patients was in quite an advanced stage and I couldn’t really hold any sort of conversation with her. (BM5061, 2013).

A key intent of the placement involved students developing recommendations for care changes arising from their assessments. Yet despite being assigned a resident by the GP tutors because of complex presentation and dementia, the absence of acute presenting symptoms were viewed as medically uninteresting. One student recounted their experience of attempting to assess two high care RACF residents with dementia:

From a medical point of view, there’s not a whole lot to do. We can take a good history, we can do a full examination and a Mini-Mental [State] Examination… but there isn’t an acute problem. My [assigned] patients… neither of them have anything really medical to talk about. They’re both very stable, it doesn’t take me very long to finish assessing them… there are no problems to manage (BM5018, 2013).

In this example, the student struggles to discern the complexity of a high-care residents’ situation. In these circumstances, it appears that some students have difficulty engaging with the complexity implicit in residents with multiple comorbidities and dementia. Arguably, such cases were seen as beneath medical interest due to the absence of acute presenting symptoms amenable to treatment.

#### Theme 2: Aged care is perceived as an unnecessary opportunity cost

The lack of capacity to undertake comprehensive medical assessments of residents was explained, in part, by these students’ attitudes towards the aged care environment. Some perceived the experience as an opportunity cost that was of little relevance for their forthcoming hospital intern year and which took time away from more important learning opportunities in the hospital. As one student noted,

[The RACF placement] wasn’t really that useful as a final year [student] in terms of the exams we’ve got coming up…we are going to be [hospital] interns next year and we need to be capable and confident in that regard (BM5005, 2013).

Indeed, a number of medical students questioned whether the clinical placement might be better positioned earlier on in the course when the pressures of pending hospital internships were less imminent: “*I think [the placement] would be of more benefit to more junior medical students in interacting with the patients and getting exposure to the vast amount of clinical signs that you may or may not get to see in the hospital setting*” (EM5041, 2013).

Further, among the 2013 cohort, this sentiment was more pronounced because time for the one-week aged care placement was taken from a clinical rotation based on student preferences (referred to as a ‘selective’). One student commented that the RACF placement reduced, “*Our selective time in the [hospital] department – neurosurgery could use me for another week* (BM5003, 2013)”. In this context, the 2013 clinical placements began with difficulty as students were displeased that hospital-based learning experiences, which they perceived as the most valuable clinical learning experiences, were considered to be lost. This further reinforced the notion of the RACF placement as an opportunity cost. Other students also perceived RACF placements as lacking challenge, being low status, and conveying a negative stigma. One student reflected, “*There is a lot of stigma with geriatrics. No one gets excited about it. I haven’t come across too many [medically trained] people that love it*” (EM5030, 2013).

Relatedly, students were challenged to conceptualise their role within the aged care setting in the absence of an established medical hierarchy, which further undermined the status of the clinical placement. For example, students in the 2013 cohort commented on the routine absence of doctors from RACFs, which they viewed as a barrier to learning. As one student noted, “*There is not a doctor here all the time and no one that we can speak to for advice on what you do or [how to] find out more information, so it’s just not the right environment*” (BM5036, 2013). Indeed, this was the first time in their undergraduate student training that no readily accessible medical supervision was available at all times. Further, reflecting a negative sentiment, even when these students identified alternative management options in the absence of a treating physician, they reported a sense of futility with respect to their capacity to influence resident care outcomes:

We come in here and we’re given our patients and we assess them, but there’s not a GP here… We have our suggestions on how management plans can be changed and we’ve picked up these problems in these patients, but there’s nothing we can do to fix them (BM5038, 2013).

Despite the intermittent presence of a GP tutor and ongoing availability of senior nurse mentors, these students considered the lack of ready access to medical staff within the RACF to be a factor that undermined the RACF as an appropriate training environment.

#### Theme 3: Aged care placements stimulate problem solving and critical reflection

Deleterious experiences and negative sentiments in the RACF context were not ubiquitous across the entire medical student cohort. While some students struggled to discern the complexity among volunteer residents and viewed the clinical placement as an opportunity cost, others saw an opportunity to extend their competency and develop capability for independent clinical decision making, particularly with respect to the management of frail elderly people with dementia. These students developed the skills to compile a comprehensive collateral history, which included thoroughly reviewing resident notes, seeking the views of interdisciplinary peers and colleagues (including nursing and paramedic students and RACF staff members), taking the time to look for non-verbal cues, and observing resident behaviour and mobility. When these students were confronted by residents with dementia (and other conditions that challenged their assessment capability), they took up the challenge to extend their knowledge and skills. One student outlined the challenge associated with gaining a more holistic understanding of residents:

I suppose [the placement] forced us to be more resourceful in how we gather clinical information… Seeing someone who doesn’t necessarily have an acute illness episode and… just trying to figure out a whole picture and ongoing generalised management plan, which considers their wishes without relying too heavily on a resident who’s not able to give you a good picture (BM5017, 2013).

Another student recounted their experience of seeking out alternative informants who could facilitate the development of a patient history. This student also recognised the value of this approach across clinical settings:

If you can’t get good history [from a person with dementia], that’s an opportunity to talk to the nurse and [to] see how much [information] you can get from them [about the resident]. Then you can also speak to their families in order to get the full picture, and so you go through a process of investigating a particular patient with dementia and seeing how you take collateral histories…That’s useful no matter where you’re working (BM5006, 2013).

When these students engaged with the challenges presented by interaction with residents with dementia, their reports suggested that they developed valuable skills that assisted them to become more resourceful clinical problem solvers. They also experienced moments of revelation evident in their surprise when confronted with the complexity of high-care RACF residents, with the associated issues of polypharmacy, comorbidity, and chronicity. One student commented, “*The most beneficial aspect of the placement was to look at polypharmacy. We were assigned some time to look at the drugs our patients were on and how they could be altered – that was quite beneficial*” (EM5044, 2013). Another reflected,

I had a good week. Not only did I get exposed to the elderly and see their issues of cognition, falls risk, depression, but I really got to see and visualise how these problems interact… So many of the residents had those issues and I learnt from that… I think it’s definitely a beneficial [placement] week and one that I’d recommend (BM5061, 2014).

In instances such as these, medical undergraduates embraced the challenges and learning opportunities inherent in the RACF placement. The challenges of aged care were reframed as cues for self-directed learning and independent clinical decision making that facilitated the development of recommendations for improving resident health and quality of life.

#### Theme 4: Contributing to resident health reinforces training and clinical decision making

With greater appreciation of the complex health issues faced by residents, students were often empowered to address problems they encountered. While all students were tasked with performing a comprehensive assessment of a volunteer resident, some recognised that they were in a position to provide a new diagnosis, revise a resident’s management plan, or make recommendations that could improve health and quality of life. One student commented, “*People are actually taking on board and thinking about using [our recommendations]. I really enjoyed that because it’s really good for us and also good for the residents*” (EM5029, 2013). Opportunities to identify and address health problems in an older-adult cohort reinforced clinical training, bedside manner, and provided a framework for independent learning and clinical decision making. One student reflected on building rapport with a resident and addressing her ongoing health concerns:

The most enjoyable [aspect of the placement] was speaking to my resident. I really got to know who she was. She really benefitted from that and I really enjoyed it. When I was speaking to her I said, “These are the medical issues that I think are important, would you like me to see what could be done to address these?” She really appreciated it, and she had some great ideas about what she’d like changed, and now it’s been given a voice [in my recommendations]… I’m really quite proud of what I found and what could potentially be put in place (EM5030, 2013).

Students also developed a new appreciation of how the clinical placement would support them in their role as hospital interns engaged with frail older patients requiring transfers to or from RACFs. One student stated that their placement experience, “*Will help a lot in getting our patients ready to be discharged to a nursing home. Now we know what facilities are available… We also know what the limitations of these facilities are”* (BM5058, 2014). Another student recounted,

Our focus [in fifth year] is on how we’d be working in the hospital, because obviously we train the majority of time in hospital and then next year we are going to be in the hospital [as interns]. It’s good to have the community [RACF] perspective because patients might be transferred to and from nursing homes under our medical and surgical teams (BM5050, 2014).

Understanding the health challenges faced by RACF residents and gaining insight into the functioning of the aged care sector imbued these students with confidence. They gained understanding and capability when conceptualising how they might collaborate and interact with RACFs as future medical practitioners and how to assess and make recommendations to improve quality of life for older people in a range of settings.

## Discussion

This paper investigated medical student experiences during a five-day clinical placement at two regional Australian RACFs. Survey data collected at the beginning of the placement indicated that most medical students had limited previous engagement with the aged care sector and commenced with an expectation that they would not enjoy the experience. The data show that less than 20 % of students were satisfied with the prospect of an aged care placement, and one in three expected to enjoy working with older adults. These findings are consistent with critiques of medical education that report a lack of interface between aged and acute care [10] and negative attitudes among medical students towards the care of older people and geriatric specialisations [18, 31, 44, 45]. Such evidence suggests that negative sentiment is widespread among medical undergraduates and not unique to the cohort of students involved in this study.

Although negative student attitudes are well documented [18, 31, 44, 45], few researchers have offered evidence to explain why such attitudes persist among medical undergraduates. Survey data from the current study concerning negative pre-placement sentiments were supported by focus-group results. Two themes emerged that provided explanation for students’ negative attitudes.

Firstly, some students struggled to engage with residents with a significant cognitive impairment, such as dementia. These students were challenged by problems with communication, lack of a clearly defined acute presentation, and the complex issues of multiple comorbidity and chronicity. These difficulties may be, in part, explained by student expectations for traditional patient-centred medicine that is challenged when a resident cannot communicate effectively or enter into a reciprocal physician-patient relationship [46]. In such circumstances, medical students may not have been adequately prepared, with appropriate clinical skills or situational awareness, to make sense of the undifferentiated comorbidities and communication challenges that characterise most frail older people resident in RACFs. The response to this situation among students who lack such skills may be, as reported in the results, to conclude that either assessment is futile in the face of seemingly irresolvable communication challenges or to infer that the resident is clinically stable thereby apparently contradicting the appropriateness of conducting an assessment. Researchers have previously reported that medical students are not adequately prepared by their curricula for communication challenges encountered with cognitively impaired patients [17–47] and that they hold perceptions of older people’s health problems as frustrating and of little clinical interest [18, 19]. Despite these sentiments, there is evidence to suggest that residents of aged care facilities do not always receive optimal health care [48–50] and can benefit from additional assessment and more targeted management, even when this is provided by students [33].

A second theme that explains negative attitudes among medical students towards aged care and older adults suggests that students conceptualised the clinical placement as an opportunity cost that reduced training time in preferred hospital settings. Some students had difficulty conceptualising the aged care environment as an appropriate setting for medical students due to the lack of an established medical hierarchy and differences from the hospital environment where they spend the majority of their placement time [9]. The study findings indicate that medical student learning is oriented around acute care environments. Such settings work to develop more than just a particular set of clinical and professional skills. They also engender particular cultural values that take root in early medical education, a process that is often referred to as the hidden curriculum [18]. The hidden curriculum describes the learning that occurs through informal interactions with students, faculty members, and medical professionals, which shapes values, attitudes, and professional identity [25]. Prominent themes that have been reported in the hidden curriculum include patient dehumanisation, disease-focussed medicine, ritualised professional identity, and emotional neutralisation [25, 51]. In some ways, the aged care placements may have exposed elements of the hidden curriculum. This is reflected in students devaluing the aged care context as a site for productive and important learning and their struggle to engage with patients who do not fit the profile of an acute presentation amenable to treatment or cure. The RACF placement was intentionally challenging. Students encountered older-adult volunteers who exhibited a range of complex cognitive and physical symptoms that moved students beyond their comfort zone and previous experiences of diagnosing and treating a single clinical problem (such as a fracture or infection). While some students struggled to engage with this level of complexity, many students indicated that they valued this experience and that it had extended their competence and increased their understanding of older-adult health issues and the aged care sector. Clinical placements in aged care settings set up the conditions to differentiate between those students who are ready to engage with the complexity of aged health care and those who remain wedded to hospital training environments with expectations for acute presentation, omnipresent medical supervision, and high status settings.

There was a clear continuum of experience among medical students, with some capable of understanding and engaging with the challenge of aged care and others who viewed the experience as confrontational and beyond their developmental needs as trainee medical professionals. Post-placement survey data indicated that positive knowledge and attitudinal changes occurred among medical students who participated in the five-day aged care placement – indicating positive placement engagement outcomes across the cohort irrespective of some negative sentiment. Medical students scored highly on the DKAT 2, indicating good knowledge of dementia before the placement, and displayed a significant increase in knowledge at the end of the placement. Changes in dementia knowledge were supported by self-reported improvements in understanding of the aged care environment among the majority of students who responded to the post-placement survey. Similarly, the survey data indicated that attitudes towards working with older adults improved significantly over the course of the clinical placement. The results of this study reinforce mixed international evidence for variable medical student attitudes towards older adults, aged care, and geriatric medicine [18–22, 45, 52]. International research shows that although students may hold negative, neutral or positive attitudes towards older adults prior to clinical engagement, exposure to this cohort as part of medical training can significantly improve attitudes [21, 45, 53]. Importantly, it has also been shown that an enduring or improved positive attitude towards older adults increases the likelihood that medical students will consider a career in geriatric medicine [22, 45]. Considering the ageing demographic profile of western societies, this highlights the imperative to provide students with positive placement experiences that overcome the perceived lack of clinical interest associated with older adults in an aged care setting.

Qualitative themes emerging from this study also highlight positive student learning experiences and meaningful encounters with vulnerable residents. Student reports indicated that the aged care placement stimulated problem solving and critical reflection about the complex health challenges in high care settings and provided opportunities for students to contribute to resident health and quality of life.

The third research theme indicates that, through student engagement in the assessment of older adults, there were powerful cues for learning that facilitated their appreciation of the complex health challenges in high-care environments. While some students were confronted and confused by non-communicative residents, an absence of an acute illness or exacerbation, and the insidious effects of chronicity, multiple comorbidities, and polypharmacy, others used the experience as an opportunity to extend their skills in accessing critical information through collateral history taking. The capacity to take a collateral history has been identified as a particularly important skill for physicians in the diagnosis and management of dementia [54]. This is because people with dementia (and other cognitive impairments) often have difficulty clearly articulating their needs (for example, thirst or pain management) and may use behaviours, bodily movements, or incoherent vocalisation in an effort to communicate [55]. Although comparatively limited research has been undertaken that has explored learning outcomes for medical students on clinical placements that include older adults, researchers have noted that attitudes towards and perceptions about this cohort improve with interpersonal contact [20]. Further, it has been hypothesised that socialisation and empathy building tasks with older adults helps to reduce the stigma of providing healthcare to this cohort and challenge perceptions of the futility of treatment [20, 56]. Researchers and medical educators who have investigated student engagement with older adults continue to recommend clinical engagement with the aged care sector as a means of improving competencies in the area of geriatric medicine [53, 57]. These findings highlight the importance of medical students having in-depth engagements with aged care residents in order to develop an informed understanding of the complexity of health care management needs beyond the hospital walls. Such skills will be critical in responding to the expansion of the older-adult population and growing prevalence of conditions such as dementia.

The fourth research theme indicated that students value the opportunity to use their training and emerging clinical decision-making skills to address the complex problems of older residents. As part of the placement, students not only performed a collaborative and comprehensive medical assessment, but also made recommendations for improving the health and quality of life of volunteer residents. These recommendations were presented to RACF staff at the end of the placement and recorded in the residents’ medical notes. This is among the most innovative aspects of the program and an approach to experiential learning that is gaining popularity in Australia. In a small-scale Western Australian study of undergraduate placements in aged care, Seaman et al. [32] reported that undergraduate medical students and their tutors valued opportunities to make contributions to the health of residents through exchanging knowledge and new techniques with RACF staff and interacting directly with vulnerable older adults. In another Australian study, Elliott et al. [33] reported that medical students (in collaboration with nursing and paramedic students) “enhance quality of care” for aged care residents by improving facility vibrancy, increasing meaningful social contact, providing opportunities for residents to engage in teaching activities, and increasing capacity for, and confidence of, care. Although embedded placement experiences in aged care settings or interactions with people with dementia remain relatively uncommon within undergraduate medical education [9, 12], community based experiential learning is considered to offer advantages over classroom and simulation-based activities. Following a systematic review of studies of experiential medical education, Doran and colleagues [58] reported that community based clinical placements support workforce acclimatisation, appropriate professional socialisation, improved patient-physician relations, and critical self-reflection concerning both biomedical and psychosocial competencies. Doran et al. [58] identified that community based placements help to vivify clinical practice and increase student understanding of how the health system functions in the real world. Exposing medical students to complex, and often difficult to discern, health issues in aged care provides them with an avenue for detailed investigation and opportunities to meaningfully improve the health-related quality of life of residents. In this way, they are able to practise and refine their clinical decision-making skills in ways that can be carried over into diverse clinical settings, such as the treatment of a person with dementia in hospital and discharge into community care. Further work is required, however, to embed aged care placements as part of ongoing medical curricula adaptations to 21^st^ century population and health system changes.

Successful learning outcomes achieved in the aged care placement were underpinned by support from RACF mentors, clinical oversight from a GP tutor, and a highly structured yet iterative placement program. Medical educators have recommended that successful pre-vocational clinical placements in RACFs rely on strong administration, internal and university support, and careful integration of staff, students, and residents within the program [32]. They also suggest that continual improvement and adaptability are important to address student expectations and emerging challenges in the learning environment [31]. The data from the current study suggests that improvements in knowledge and attitudes may be achieved among some medical students following five days of embedded clinical training. Although positive outcomes have been achieved in this placement, the persistence of negative attitudes and encounters among some students suggests that more work is required to refine the placement structure in the future and, more importantly, to address a pervasive culture in medical education that devalues aged care and older adults as irrelevant or unworthy of the attention of medical students. When medical student placements have been embedded as part of curricula in the United States, for example, educators noted a political backlash citing negative perceptions among senior faculty staff (viewed as taking time away from important internal medicine placements) and the transmission of negative attitudes within learner cohorts [31]. Such embedded attitudes among teaching staff and students may also relate to limited resources for teaching and assessing geriatric medicine competencies where curricula focus predominantly on biomedical knowledge and hospital-based medicine [53]. Powers et al. [31] indicate that strong leadership and support from within medical schools may ultimately be the critical factor in ensuring the success of aged care clinical placements.

### Limitations and future research opportunities

There are few measures of dementia knowledge that can reliably gauge understanding of the syndrome among cohorts with a high level of education. Potential ceiling effects in the DKAT 2 may limit the extent to which pre- and post-placement knowledge differences can be reliably ascertained. Limitations in the measure used in this study potentially underestimate knowledge change due to a lack of sensitivity. Further work is required to develop a measure of dementia knowledge that is sensitive enough to measure knowledge change among highly educated individuals in the health sector. A recently published dementia knowledge scale developed by members of the research team (MA and AR) will address this gap in future studies [59].

This study was also preliminary in nature and the scale of investigation was limited to two RACFs that hosted medical students from a southern Tasmanian Clinical School during 2013 and 2014. Future national expansion of the TACF program would be desirable to attain a nationally representative sample. As all students were from the same Clinical School, it is not yet possible to determine the extent to which different undergraduate medical curricula and cultures influence student engagement.

In this research, students were embedded in RACFs for five days and well supported by an onsite University medical tutor and senior mentors (experienced nurses and care staff) from the participating facilities. The long-standing nature of the clinical placement program (which began in 2011) and high level of faculty and RACF support and supervision arguably provided an optimal learning experience. However, it is possible that replication of these placements in the context of a less supportive environment, where staff members may exhibit negative attitudes towards students or medical tutors could not routinely attend the placements, may be a threat to effectiveness of such learning opportunities. In RACFs, there is currently limited engagement of GPs with residents and few permanent appointments in this sector [60, 61], which could undermine student perceptions about the relevance of the placement. This situation, however, does not reflect a lack of need or complexity in aged care, but rather the traditional role of the GP in the community (where they are often based in clinics or practices). In this respect, it was necessary to have a GP tutor onsite each day to supervise and direct student activities as well as helping them to understand the relevance of the placement at a time when GP roles are altering with demographic changes (including population ageing). The GP tutor was not present at all times, allowing students to develop their independent clinical decision making, but they were available at the end of each day to support and contextualise the placement experience. Researchers in other institutions should take care to ensure that placement programs are well designed with sufficient engagement from the aged care sector and educational institution support to achieve an optimal mix of independent learning and supervision.

The placement of students in RACFs also has the opportunity to elicit impacts beyond student learning and influence care quality for residents and changes in the clinical behaviour of staff. While a small number of studies have reported the positive impacts of health student placements on resident quality of life [32, 33], few have investigated how students potentially change the clinical practice of RACF staff. It is possible that embedding fifth-year medical students (who have a high level of training in such topics as pharmaceuticals, pain management, and diagnosis) may bring a greater level of awareness to the aged care sector about the need to provide best-evidence care – even towards the very end of life. This is an area of potential future investigation.

## Conclusion

Clinical placements in RACFs challenge medical students to become more resourceful and independent in their assessment and decision making with vulnerable older adults. While not all students can be highly engaged with the learning opportunities over a relatively short five-day placement, our mixed methods findings suggest that many find the experience to be rewarding and relevant in the context of their undergraduate training and preparations for hospital internship. Critical elements of a successful placement include a high degree of structure, support and supervision from medical tutors and senior RACF staff, and an iterative approach to program design that address emergent challenges and student concerns. Remaining challenges include inculcating positive attitudes among medical undergraduates towards the aged care sector and older adult health (including dementia) and achieving a better balance between acute and non-acute training opportunities. Considering that the placement program described in this paper is arguably the most intensive and large-scale engagement with the aged care sector and older-adult health that Australian undergraduate medical students have within a five-year undergraduate degree, there appears to be a horizon of opportunity to make inroads into knowledge, attitudes, and clinical competence by increasing experiential education in this sector.

## Abbreviations

DKAT 2: Dementia Knowledge Assessment Tool Version 2.
GP: General Practitioner
RACF: Residential Aged Care Facility
TACFP: Teaching Aged Care Facilities Program
